# The clinical efficacy of Astragalus-containing Chinese patent medicines in the effective treatment of heart failure: A systematic review and network meta-analysis

**DOI:** 10.1097/MD.0000000000049013

**Published:** 2026-05-29

**Authors:** Fangfang Rui, Yunfeng Di, Chun Li, Tianhua Liu, Yan Wu

**Affiliations:** a School of Traditional Chinese Medicine, Beijing University of Chinese Medicine, Beijing, China; b Modern Research Center for Traditional Chinese Medicine, School of Chinese Materia Medica, Beijing University of Chinese Medicine, Beijing, China; c Key Laboratory of TCM Syndrome and Formula (Beijing University of Chinese Medicine), Ministry of Education, Beijing, China.

**Keywords:** Astragalus, clinical efficacy, heart failure, network meta-analysis, traditional Chinese medicine

## Abstract

**Background:**

The incidence of cardiovascular diseases, particularly heart failure, has been rising year by year. Traditional Chinese medicines containing Astragalus have been widely used in clinical research. However, there is no definitive research on the efficacy of proprietary Chinese medicines containing Astragalus. A network meta-analysis was conducted to investigate the efficacy and safety of oral traditional Chinese medicines containing Astragalus for the treatment of heart failure.

**Methods:**

This study searched 6 databases for clinical randomized controlled trials involving the use of traditional Chinese herbal medicines containing Astragalus alone or in combination with other drugs, with a search period from the establishment of the databases to May 27, 2025. Using the Cochrane Quality Assessment Manual, we conducted a bias analysis of the included studies, and the extracted data were analyzed using network meta-analysis in Stata 18.

**Results:**

A total of 18 studies were included, involving 1584 patients with heart failure, with 16 types of traditional Chinese medicines containing Astragalus. The network meta-analysis via Surface Under the Cumulative Ranking Curve analysis indicated that: Compared to using Western medicine (WM) alone, Astragalus Granule + WM ranked best for improving overall clinical efficacy rate; Qiangxin Capsule + WM was optimal for improving six-minute walk test; Astragalus Granule + WM showed the best efficacy for reducing B-type natriuretic peptide levels; Yanxing Decoction + WM ranked first for lowering N-terminal pro-B-type natriuretic peptide levels; Xinlishen Compound + WM was the optimal combination for improving left ventricular ejection fraction; Qili Qiangxin Capsule + WM performed best for reducing left ventricular end-diastolic diameter; Yangxin Tongmai II Prescription + WM exhibited the highest efficacy for decreasing Minnesota Heart Failure Quality of Life Questionnaire scores.

**Conclusions:**

Adding oral traditional Chinese medicine containing Astragalus to WM treatment can further improve the clinical efficacy of heart failure. However, due to the limited number and quality of the included studies in this research, the above conclusions still require further validation through well-designed randomized double-blind controlled trials.

## 1. Introduction

Heart failure (HF) is a complex clinical syndrome characterized by symptoms and signs resulting from any structural or functional abnormalities in cardiac diastolic and systolic function. The etiology of these abnormalities is primarily attributed to reduced cardiac output or elevated intracardiac pressure, resulting from structural and functional cardiac abnormalities. These abnormalities have a significant impact on patients’ quality of life and prognosis.^[1,2]^ According to the latest statistics, there are over 64 million people with heart failure worldwide, with a case-fatality rate as high as 50% after diagnosis. Despite the stabilization or decline in incidence rate observed in industrialized countries, the prevalence of the condition continues to rise. For instance, in the United States, it is estimated that the prevalence of HF will increase by 46% from 2012 to 2030, while concurrently medical costs will rise by 127%.^[3,4]^ The prevalence of HF has increased over time and is projected to continue rising over the next decade, from 2.4% of the total population in 2012 to 3.0% by 2030.^[5]^ Although modern Western medicine (WM) treatments (such as beta-blockers, angiotensin converting enzyme inhibitors/angiotensin receptor blockers, angiotensin receptor neprilysin inhibitors, and sodium-dependent glucose transporter 2 inhibitors) have significantly improved patient survival rates, some patients still experience poor symptom control, repeated hospitalizations, and adverse drug reactions.^[1]^ Therefore, exploring integrated traditional Chinese medicines and WM treatment protocols holds significant clinical importance.

Astragalus membranaceus, traditional Chinese medicines is known as a “key tonic.” Astragalus has a sweet and warm nature, with effects including tonifying qi and nourishing deficiency, promoting diuresis and reducing edema, and invigorating heart yang; traditional Chinese medicines classifies HF under categories such as “dyspnea” and “palpitations,” with the core pathogenesis being deficiency of heart yang and impaired blood circulation, leading to retention of water and dampness and stasis of blood. Astragalus contains active components such as flavonoids and saponins. It enhances immune function, reduces myocardial cell apoptosis, improves mitochondrial function, and alleviates cardiac insulin resistance, thereby exerting an anti-heart failure effect.^[6,7]^ The effects of Astragalus align with this pathogenesis.^[8]^ Various Astragalus-containing traditional Chinese medicine formulations (such as Astragalus and Lycium Capsules, Astragalus Injection, and Ginseng and Astragalus Tonifying Injection) have been widely used as adjunctive therapies for HF in recent years.^[9]^ Clinical observations suggest that these herbs may have a greater effect when used alongside conventional Western medications. However, it is unclear whether different herbal formulations have different levels of efficacy and safety.

Currently, although several randomized controlled trials have explored the efficacy of combing different Astragalus preparations with Western medicine for the treatment of HF,^[10]^ traditional meta-analyses have difficulty systematically comparing these interventions due to heterogeneity in study design, treatment regimens, and evaluation criteria. Network meta-analysis is an emerging statistical method that can integrate direct and indirect comparative evidence in order to quantitatively rank multiple interventions.^[11]^

This aim of this study to use the network meta-analysis method to systematically evaluate the efficacy and safety of different Astragalus-containing traditional Chinese medicines formulations combined with conventional WM for the treatment of HF. The goal is to provide evidence-based guidance for the rational use of drugs in clinical practice.

## 2. Methods

### 2.1. Protocol and registration

The protocol for this network meta-analysis was registered with the International Prospective Register of Systematic Review (No. CRD420251079736).

### 2.2. Inclusion and exclusion criteria

Patients with a clear diagnosis of HF, with no restrictions on age, gender, or ethnicity. Interventions: the control group was treated with WM, including hydrochlorothiazide, benazepril, spironolactone, and other conventional Western drugs. The control group was treated with a combination of WM and Chinese patent medicine containing Astragalus membranaceus. There were no restrictions on the duration of treatment. Outcome measures: clinical overall response rate, left ventricular ejection fraction (LVEF), NTpro-BNP, B-type natriuretic peptide (BNP), six-minute walk test (6MWT), left ventricular end-diastolic diameter (LVEDD), Minnesota Heart Failure Quality of Life Questionnaire (MLHFQ), etc. Exclusion criteria: non-English or Chinese literature; literature with incomplete data and unable to obtain original data for analysis; reviews, biological experiment literature, research plans, etc., as well as duplicated publications; clinical research literature combining other diseases.

### 2.3. Search strategy

Computerized searches were conducted in English using databases such as PubMed, Web of Science, and the Cochrane Library, and in Chinese using databases such as China National Knowledge Infrastructure, Wanfang Database, and VIP Database. A combination of subject terms and free-text keywords was used. Chinese search terms included Astragalus; Astragalus preparations; randomized controlled trials; control; random; HF, left heart failure, right heart failure, HF, congestive heart failure, English search terms include Astragalus propinquus, randomized controlled trials, HF, etc, with a time limit from the establishment of the database to May 27, 2025.

### 2.4. Literature screening and data extraction

The obtained literature was imported into Endnote software for screening, and duplicate documents were excluded. Then, based on the established inclusion and exclusion criteria, the researchers independently screened, evaluated, and extracted information from the included literature. The extracted content included article titles, authors, years, baseline conditions, intervention measures, outcome indicators, and treatment times. If there were any disagreements or discrepancies, a third researcher would reevaluate the information.

### 2.5. Literature quality evaluation

The quality of the included studies was assessed using the Cochrane Collaboration’s risk assessment tool, focusing on several key aspects: random allocation method, use of blinding, concealment of allocation, completeness of outcome data, selective reporting, and other sources of bias. For each assessment criterion, “low risk” wash assigned if the information was complete and accurate, “high risk” if it was incomplete or inaccurate, and “unclear risk” if the information was insufficient. The full text was read, and 2 professionals conducted cross-checking of the information to complete the quality assessment.

### 2.6. Data integration and analysis

Statistical analysis was performed using Stata 18.0 software (StataCorp LLC, College Station). For categorical data, the odds ratio was used as the effect measure. For continuous data, the mean difference was used as the effect measure. When units were not consistent, the standardized mean difference was used as the combined effect measure, with 95% confidence intervals used for interval estimation. A comparison-correction funnel plot was used to identify small-sample effects between studies and assess publication bias. For outcome measures, Surface Under the Cumulative Ranking Curve (SUCRA) was used to rank the efficacy of each intervention. The advantages and disadvantages of each drug as the optimal treatment were evaluated.

## 3. Results

### 3.1. Literature search

**Figure 1.**
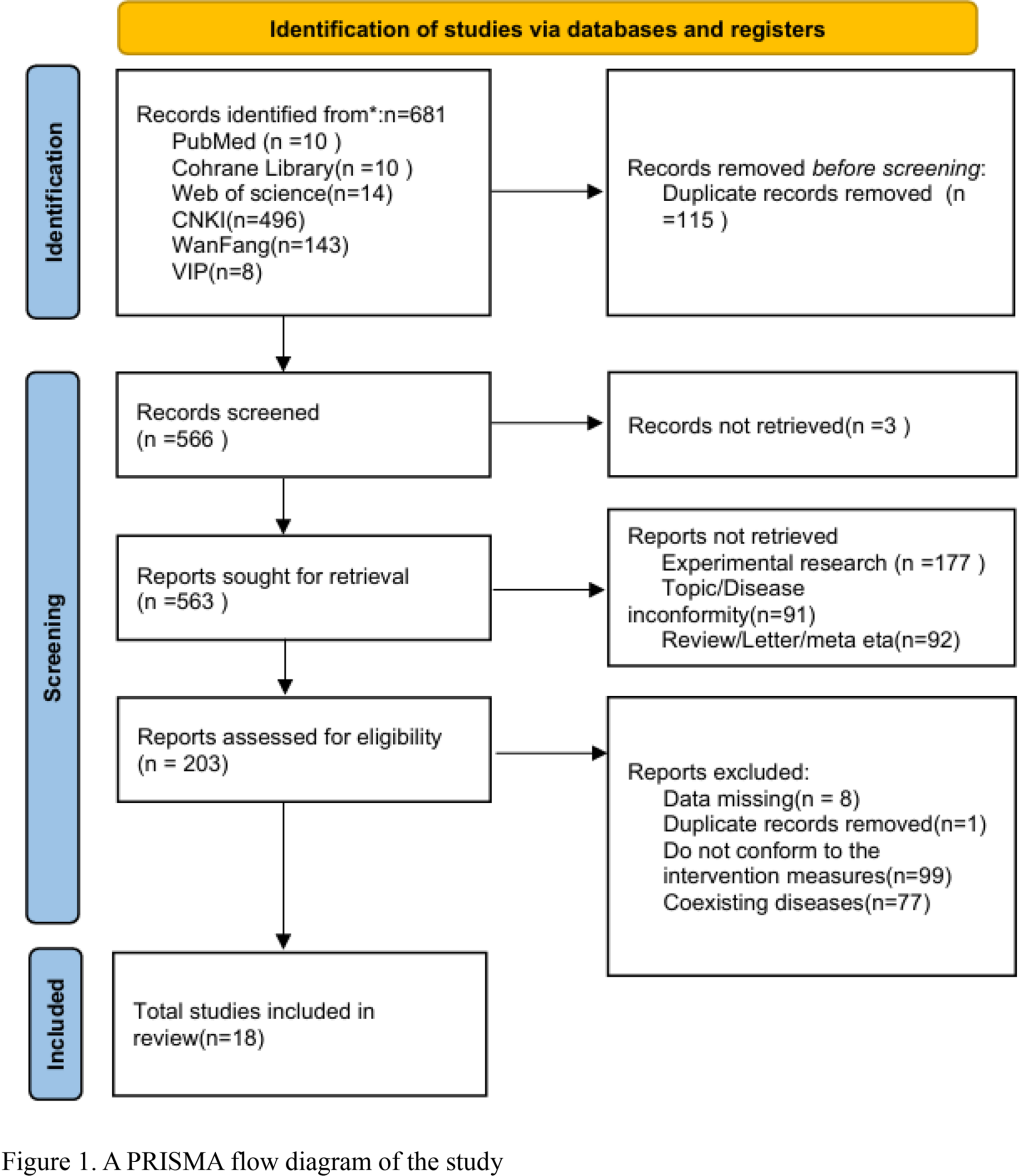
A PRISMA flow diagram of the study.

The network meta-analysis identified a total of 681 relevant original studies, including 34 English-language studies and 647 Chinese-language studies, involving 16 types of oral traditional Chinese medicine formulations containing Astragalus, including Yixin Paste, Qili Qiangxin Capsule, Xinlishen Compound, Astragalus Granule, Qiangxin Capsule, Yixintong Capsule, Shencao Tongmai Granules, Kangda Xin Oral Liquid, Yangxin Tongmai II Prescription, Yanxing Decoction, Qiangxinkechuan Decoction, Guizhi Yangxin Capsule, Yiqi Qiangxin Liquid, and Astragalus Tetrandrae Decoction plus Ephedra combined with WM. First, 115 duplicate articles were excluded. Three articles had no original text. After reading the titles and abstracts, 360 studies were excluded. Among the remaining 203 studies, 18 met the inclusion criteria, as shown in Figure 1.

### 3.2. Inclusion of basic information from the literature

A total of 18 studies were included, with 6 trials^[12–17]^ discussing the clinical overall efficacy rate of traditional Chinese medicines formulations containing Astragalus combined with WM for the treatment of HF. The main oral traditional Chinese medicines formulations included Qili Qiangxin Capsule, Shencao Tongmai Granules, Qiangxin Capsule, Kangda Xin Oral Liquid, Astragalus Granule, and Yiqi Qiangxin Liquid.

Have 7 trials^[14,16,18–22]^ explored the 6MWT of oral traditional Chinese medicines formulations containing Astragalus in the treatment of HF, including Qili Qiangxin Capsule, Shencao Tongmai Granules, Qiangxin Capsule, Yanxing Decoction, Astragalus Granule, and Astragalus Tetrandrae Decoction plus Ephedra.

Have 6 trials^[16,18,19,23–25]^ assessed the levels of BNP in patients with HF treated with traditional Chinese medicines formulations containing Astragalus, including Yixin Paste, Qili Qiangxin Capsule, Qiangxin Capsule, Astragalus Granule, Guizhi Yangxin Capsule, and Xinlishen Compound.

Have 7 trials^[13,17,20,22,26–28]^ evaluated the effect of Chinese herbal medicines containing Astragalus on N-terminal pro-brain natriuretic peptide (NT-proBNP) levels in patients with HF, including Xinlishen Compound, Qili Qiangxin Capsule, Yangxin Tongmai II Prescription, Yanxing Decoction, Astragalus Tetrandrae Decoction plus Ephedra, Qiangxinkechuan Decoction, and Yiqi Qiangxin Liquid.

Have 11 trials^[13,16,18–21,23–26,28]^ evaluated the LVEF of Chinese patent medicines containing Astragalus in the treatment of HF, including Yixin Paste, Qili Qiangxin Capsule, Qiangxin Capsule, Astragalus Granule, Guizhi Yangxin Capsule, Xinlishen Compound, and Qiangxinkechuan Decoction.

Have 3 trials^[14,27,29]^ evaluated the MLHFQ, including Yangxin Tongmai II Prescription, Shencao Tongmai Granules, and Danqi baoxinfang.

Have 4 trials^[13,18,20,24]^ assessed LVEDD levels, including Astragalus and Salvia Cardiac Strengthening Capsules, Yangshen Tang Jiawei Granules, and Cinnamon Twig Nourishing Heart Capsules. There were no statistically significant differences in baseline levels between groups. Baseline levels are shown in Table 1.

**Table 1:** Basic characteristics of Chinese patent medicines containing Astragalus membranaceus included in the literature for the treatment of heart failure.

Study ID	Sample size (T/C)	Gender (male/female	Mean/median age		Course of disease (y/m)		Treatment		Duration	Outcome details
			T	C	T	C	T	C		
Gao et al^[12]^	233 (118/115)	132/101	60.6	60.8	2–6	1.4–6.3	YXTJN + WM	WM	6 w	①
Cheng WJ^[13]^	172 (88/84)	94/78	67.1 ± 9.7	68.9 ± 11.6	9.7 ± 3.8	10.4 ± 4.1	QLQXJN + WM	WM	12 w	①②④⑥
Li^[14]^	160 (80/80)	64/96	65.82 ± 6.83	64.04 ± 7.74	–		SCTMKL + WM	WM	12 w	①⑤⑦
Liu^[15]^	76 (38/38)	37/39	45.18 ± 13.1	49.18 ± 12.48	–		KDXKFY + WM	WM	2 w	①
Xiao^[16]^	92 (46/46)	50/42	63.21 ± 5.05	63.35 ± 5.27	–		HQKL + WM	WM	4 w	①②③⑤
Zuo^[17]^	60 (30/30)	30/30	60–74		–		YQQXY + WM	WM	8 w	①④
Feng^[18]^	42 (21/21)	25/17	55.6 ± 10.32	54.66 + 10.4	–		QLQXJN + WM	WM	24 w	②③⑤⑥
Li^[19]^	65 (34/31)	37/38	70.21 ± 5.32	69.97 ± 5.36	44.56 ± 8.85	41.87 ± 5.67	QXJN + WM	WM	2 w	②③⑤
Pan QJ^[20]^	60 (30/30)	27/33	66.47 ± 13.90	60.20 ± 13.48	5.43 ± 1.77	5.93 ± 2.78	YXTJWKLJ + WM	WM	4 w	②④⑤⑥
Yang and Lu^[21]^	45 (22/23)	25/20	63 ± 4.7	62.5 ± 4.3	5.6 ± 3.4	5.9 ± 4.3	HQKL + WM	WM	2 w	②⑤
Zhao^[22]^	60 (30/30)	25/35	60.73 ± 9.229	59.73 ± 8.769	3.08 ± 1.526	3.17 ± 1.918	FJHQTJMHNBFKL + WM	WM	4 w	④⑤
Chen SH^[23]^	60 (30/30)	35/25	66.71 ± 8.24	64.19 ± 9.04	1–18	6–15	YXG + WM	WM	3 w	②③
Song^[24]^	60 (30/30)	37/23	60.65 ± 7.65	59.32 ± 8.34	6.89 ± 5.64	7.58 ± 5.32	GZYXJN + WM	WM	4 w	②③⑥
Wei et al^[25]^	80 (40/40)	48/32	68.7 ± 4.7	69.2 ± 4.5	7.8	8.1	XLSHJ + WM	WM	12 w	②③
Huang^[26]^	60 (30/30)	38/22	74.83	74.63	–		XLSHJ + WM	WM	4 w	②④
Lu^[27]^	108 (54/54)	60/48	66.11 ± 8.11	64.33 ± 9.17	7.17 ± 3.25	7.87 ± 3.30	YXTM II HF + WM	WM	2 w	④⑦
Qu^[28]^	91 (46/45)	47/44	63.4 ± 7.9	63.7 ± 9.2	–		QXKCTKLJ + WM	WM	1 w	②④
Li^[29]^	60 (30/30)	29/31	72.7 ± 6.3	72.7 ± 5.2	–		DQBXF + WM	WM	4 w	⑦

Outcome details: ① Clinical efficacy rate; ② LVEF; ③ BNP; ④ NT-proBNP; ⑤ 6MWT; ⑥ LVEDD; ⑦ MLHFQ.

*Notes*: Qili Qiangxin Capsule: QLQXJN, Shencao Tongmai Granules: SCTMKL, Yixintong Capsule: YXTJN, Kangda Xin Oral Liquid: KDXKFY, Astragalus Granule: HQKL, Yiqi Qiangxin Liquid: YQQXY, Qiangxin Capsule: QXJN, Yanxing Decoction: YXTJWKLJ, Astragalus Tetrandrae Decoction plus Ephedra: FJHQTJMHNBFKL, Yixin Paste: YXG, Guizhi Yangxin Capsule: GZYXJN, Xinlishen Compound: XLSHJ, Yangxin Tongmai II Prescription: YXTM II HF, Qiangxinkechuan Decoction: QXKCTKLJ, Danqi baoxinfang: DQBXF, Western medicine: WM.

6MWT = six-minute walk test, BNP = B-type natriuretic peptide, LVEDD = left ventricular end-diastolic diameter, LVEF = left ventricular ejection fraction, MLHFQ = Minnesota Heart Failure Quality of Life Questionnaire, NT-proBNP = N-terminal pro-brain natriuretic peptide.

### 3.3. Literature quality evaluation

**Figure 2.**
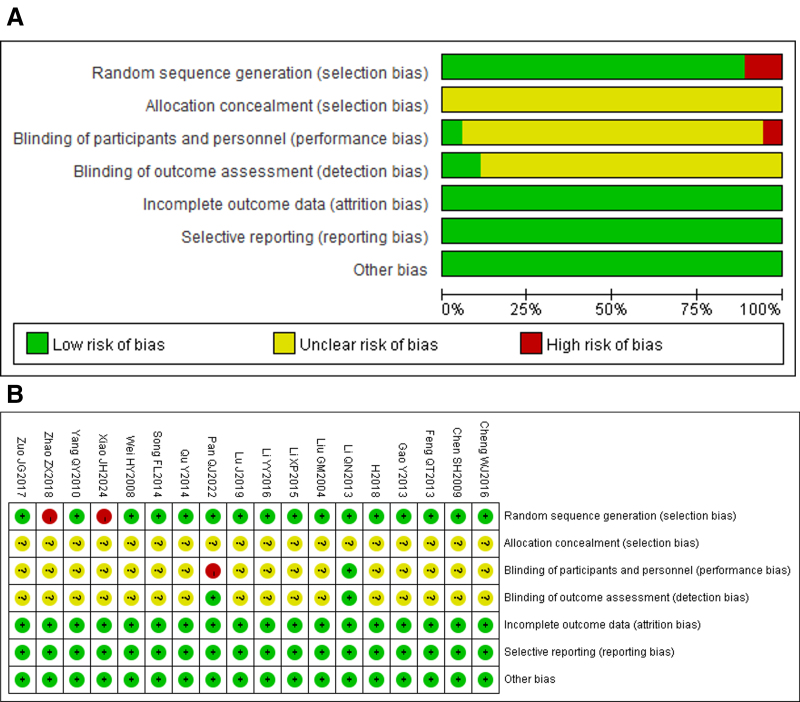
Risk of bias in all studies included in the review. (A) Risk of bias graph; (B) risk of bias summary.

All 16 studies mentioned the use of randomization methods and were therefore rated as “low risk”; 2 studies did not use randomization methods and were rated as “high risk”; 18 studies did not describe allocation concealment and were rated as “unclear”; one study used a double-blind method and was rated as “low risk”; one study did not use a blind method and was rated as “high risk”; the remaining studies did not mention blind methods and were rated as “unclear”; all included studies had complete data, so they were rated as “low risk”; all studies reported the observed outcomes, so they were rated as “low risk”; none of the included studies identified other biases, so they were rated as “low risk,” as shown in Figure 2.

### 3.4. Evidence network

**Figure 3.**
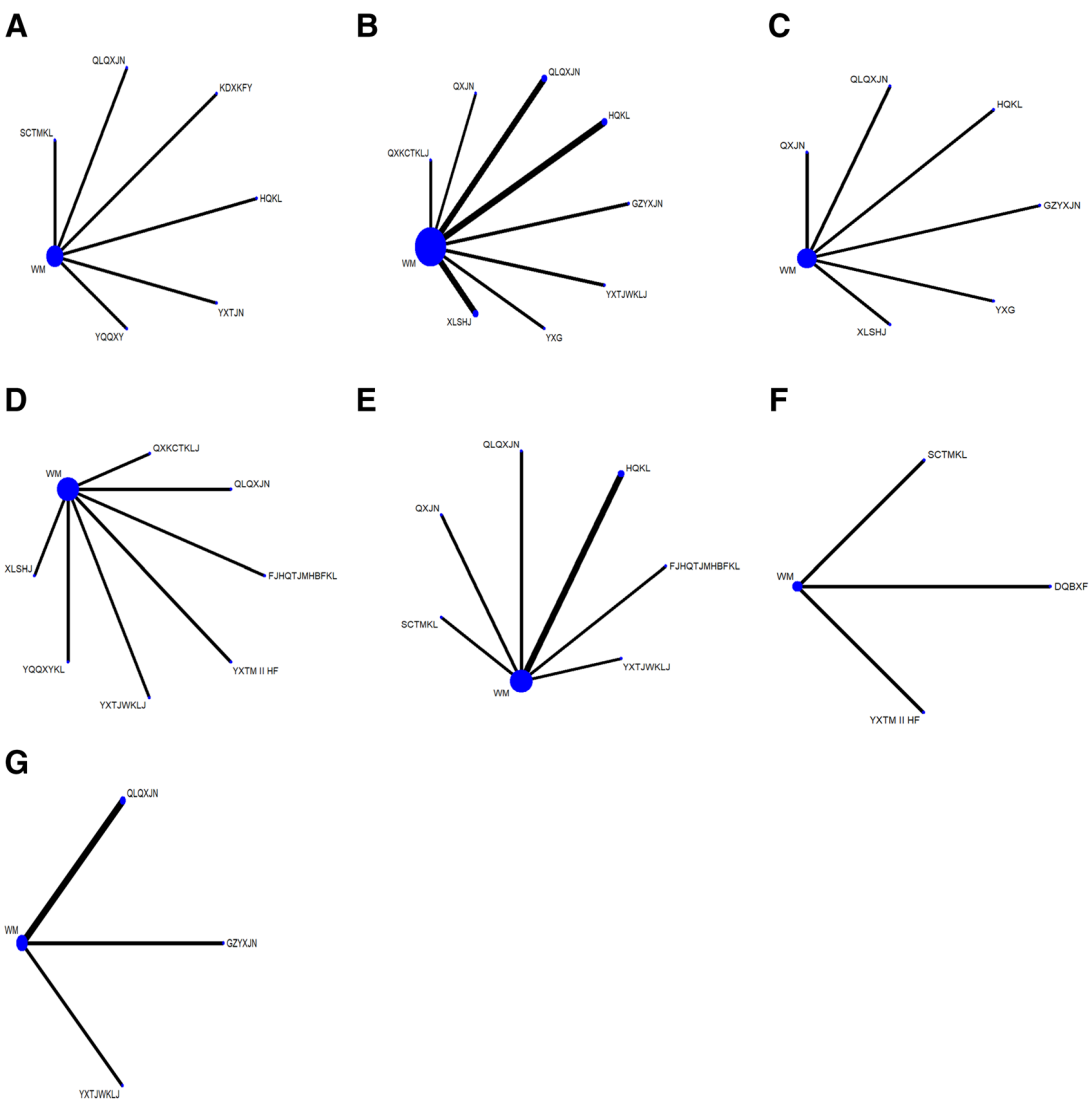
Network evidence map of outcomes of traditional Chinese medicine containing Astragalus membranaceus for the treatment of heart failure. (A) Clinical efficacy rate, (B) LVEF, (C) BNP, (D) NT-proBNP, (E) 6MWT, (F) MLHFQ, (G) LVEDD. *Notes*: Qili Qiangxin Capsule: QLQXJN, Shencao Tongmai Granules: SCTMKL, Yixintong Capsule: YXTJN, Kangda Xin Oral Liquid: KDXKFY, Astragalus Granule: HQKL, Yiqi Qiangxin Liquid: YQQXY, Qiangxin Capsule: QXJN, Yanxing Decoction: YXTJWKLJ, Astragalus Tetrandrae Decoction plus Ephedra: FJHQTJMHNBFKL, Yixin Paste: YXG, Guizhi Yangxin Capsule: GZYXJN, Xinlishen Compound: XLSHJ, Yangxin Tongmai II Prescription: YXTM II HF, Qiangxinkechuan Decoction: QXKCTKLJ, Danqi baoxinfang: DQBXF. 6MWT = six-minute walk test, LVEDD = left ventricular end-diastolic diameter, MLHFQ = Minnesota Heart Failure Quality of Life Questionnaire.

The results of the evidence network showed that 6 studies reported improvements in overall clinical efficacy, involving 6 traditional Chinese medicines, forming 6 direct comparisons; 11 studies reported LVEF, involving 8 traditional Chinese medicines, forming 8 direct comparisons; 6 studies reported BNP, involving 6 traditional Chinese medicines, forming 6 direct comparisons; and 7 studies reported NT-proBNP, involving 7 traditional Chinese medicines, forming 7 direct comparisons; 7 studies reported on 6MWT, involving 6 traditional Chinese medicines formulations, forming 6 direct comparisons; 3 studies reported on MLHFQ, involving 3 traditional Chinese medicines formulations, forming 3 direct comparisons; 4 studies reported on LVEDD, involving 3 traditional Chinese medicines formulations, forming 3 direct comparisons. The vertices in the network evidence diagram represent different intervention methods, and no closed loops were formed between the interventions; the lines connecting the nodes represent the number of randomized controlled trials studies included, with the thickness of the lines proportional to the number of related studies. As demonstrated in Figure 3, the group combining traditional Chinese medicines with conventional Western medicine was compared with the conventional Western medicine group.

### 3.5. Inconsistency test

Since none of the 7 outcome measures included in this study formed a closed loop, there was no need to perform inconsistency testing.

### 3.6. Publication bias funnel plot

**Figure 4.**
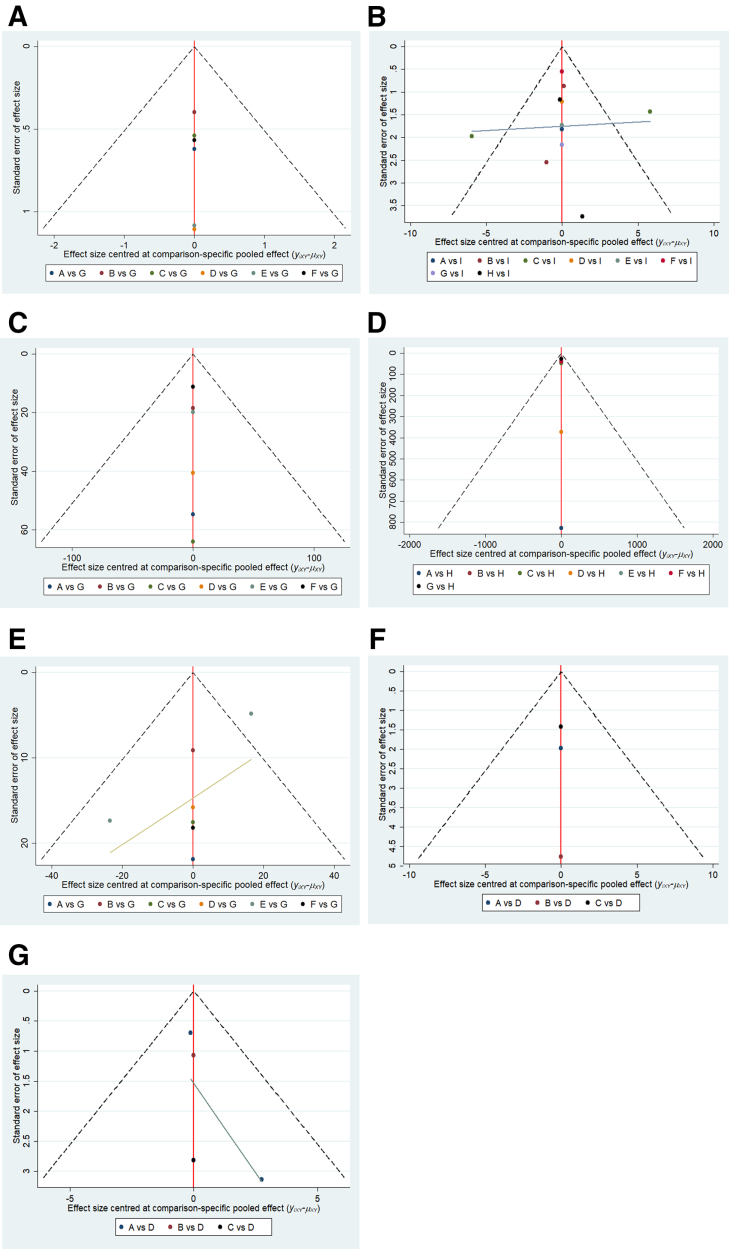
Funnel plot of outcomes of Chinese patent medicines containing Astragalus membranaceus for the treatment of heart failure. (A) Clinical efficacy rate, (B) LVEF, (C) BNP, (D) NT-proBNP, (E) 6MWT, (F) MLHFQ, (G) LVEDD. 6MWT = six-minute walk test, LVEDD = left ventricular end-diastolic diameter, LVEF = left ventricular ejection fraction, MLHFQ = Minnesota Heart Failure Quality of Life Questionnaire, NT-proBNP = N-terminal pro-brain natriuretic peptide.

Funnel plots were constructed for each outcome measure included in the study. The results showed that the funnel plots for the overall clinical response rate and MLHFQ were reasonably symmetrical, indicating minimal publication bias. The funnel plots for the remaining outcome measures were asymmetrical, suggesting the possibility of small-sample effects or publication bias among studies. Most data were symmetrically distributed on both sides of the zero line, while only a portion of the data were more dispersed and showed a skewed distribution, indicating the presence of some degree of publication bias and small-sample effects (Fig. 4).

### 3.7. Results of a network meta-analysis of traditional Chinese medicine containing Astragalus membranaceus for the treatment of heart failure

#### 3.7.1. Clinical efficacy rate

**Figure 5.**
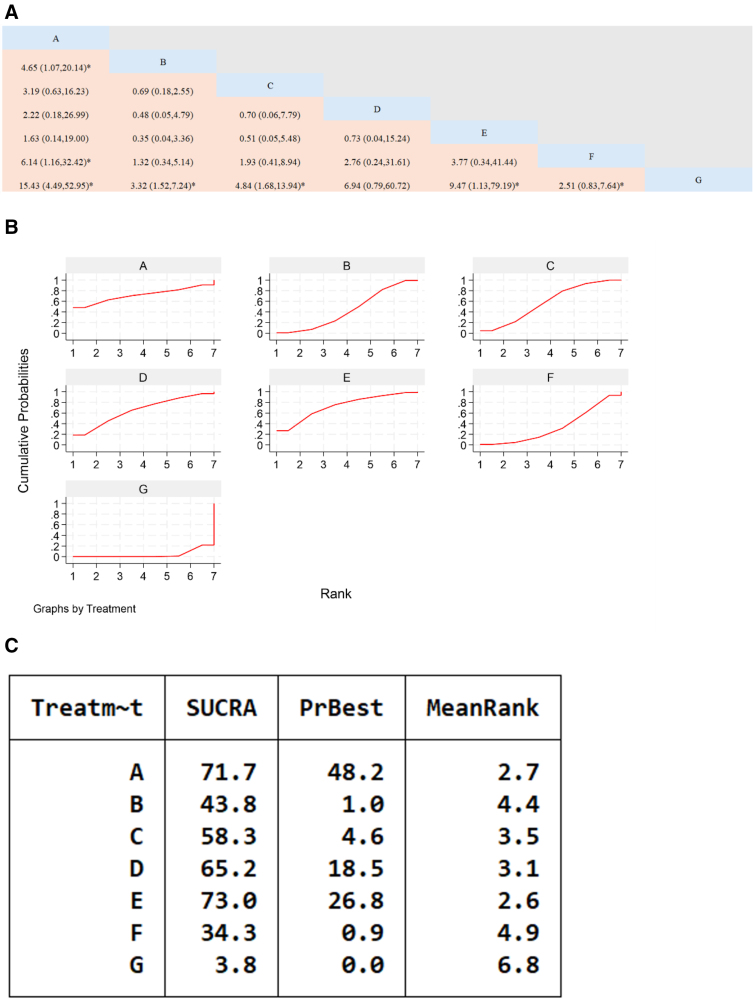
Network meta-analysis of the efficacy of Chinese patent medicines containing Astragalus membranaceus in the treatment of heart failure. (A) The league table results of the pairwise network comparisons for improvements in clinical efficacy rate; (B) SUCRA plot of changes in clinical efficacy rate; (C) surface under the cumulative ranking. (A) Yixintong Capsule; (B) Qili Qiangxin Capsule; (C) Shencao Tongmai Granules; (D) Kangda Xin Oral Liquid; (E) Astragalus Granule; (F) Yiqi Qiangxin Liquid; (G) WM. SUCRA = Surface Under the Cumulative Ranking Curve, WM = Western medicine.

Seven studies reported the overall clinical efficacy rate, involving 6 types of traditional Chinese medicine formulations and a total of 781 patients. The results of the network meta-analysis showed that the combination of Yixintong Capsule, Qili Qiangxin Capsule, Shencao Tongmai Granules, Astragalus Granule, and Yiqi Qiangxin Liquid with WM treatment was superior to the use of WM alone, with statistically significant differences (*P* < .05); Comparisons among traditional Chinese medicines formulations indicated that Yixintong Capsule combined with WM were more effective than Qili Qiangxin Capsule combined with WM and Yiqi Qiangxin Liquid combined with WM, with statistically significant differences (*P* < .05). Comparisons among the remaining traditional Chinese medicines formulations showed no statistically significant differences, as shown in Figure 5.

The cumulative ranking probability chart shows that the specific ranking of SUCRA for the improvement of efficacy of Chinese patent medicines containing Astragalus is as follows: Astragalus granules + WM > Yixintong Capsule + WM > Kangda Xin Oral Liquid + WM > Shengcao Tongmai Granules + WM > Qili Qiangxin Capsule + WM > Yiqi Qiangxin Liquid + WM > WM.

#### 3.7.2. Six-minute walk test

Seven studies reported on the 6MWT, involving 6 types of traditional Chinese medicines, with a total of 512 patients. The results of the NMA showed that the combination of Qiangxin capsule and Astragalus Granule with WM was more effective than WM alone in improving patients’ 6MWT, with a statistically significant difference (*P* < .05). There were no other statistically significant differences, and there were no statistically significant differences between the different types of traditional Chinese medicines.

**Figure 6.**
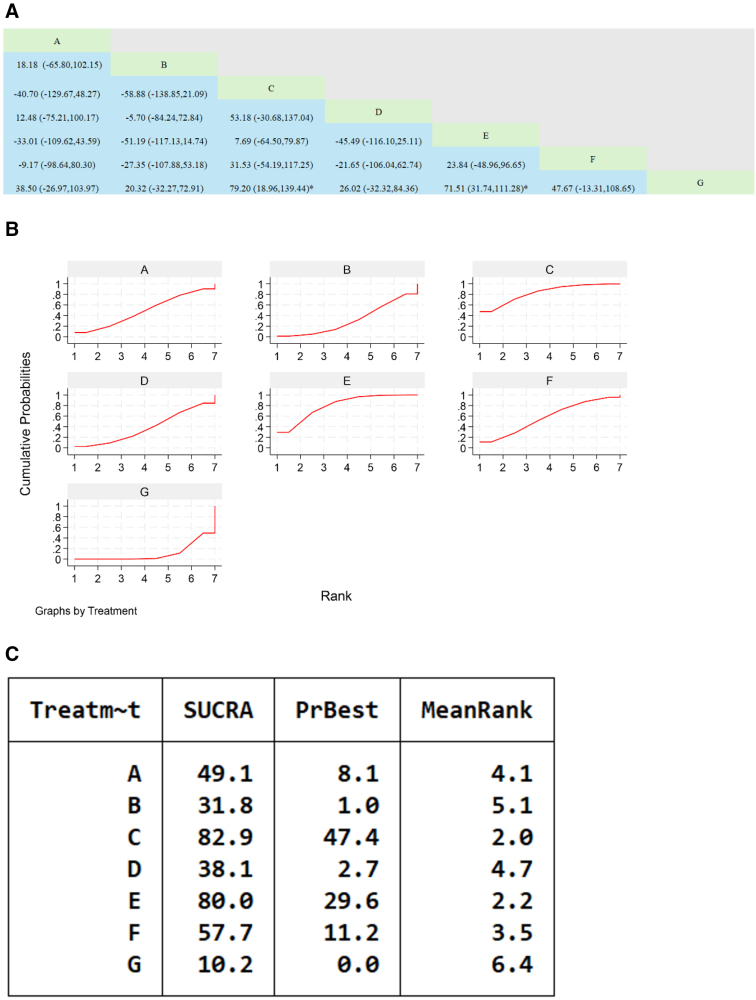
A network meta-analysis of Chinese patent medicines containing Astragalus membranaceus improving the 6MWT of patients with heart failure. (A) The league table results of the pairwise network comparisons for improvements in 6MWT; (B) SUCRA plot of changes in 6MWT; (C) surface under the cumulative ranking. (A) Qili Qiangxin Capsule; (B) Shencao Tongmai Granules; (C) Qiangxin Capsule; (D) Yangxin Decoction; (E) Astragalus Granule; (F) Astragalus Tetrandrae Decoction plus Ephedra; (G) WM. 6MWT = six-minute walk test, SUCRA = Surface Under the Cumulative Ranking Curve, WM = Western medicine.

Cumulative ranked probability plot showing the specific ranking of SUCRA for traditional Chinese medicines containing Astragalus in improving the 6MWT: Qiangxin Capsule + WM > Astragalus Granule + WM > Astragalus Tetrandrae Decoction plus Ephedra + WM > Qili Qiangxin Capsule + WM > Yanxin Decoction + WM > Shencao Tongmai Granules + WM > WM, as shown in Figure 6.

#### 3.7.3. B-type natriuretic peptide

Six studies reported on the assessment of BNP levels, involving 6 types of traditional Chinese medicines, with a total of 399 patients. The results of the network meta-analysis showed that Yixin Paste, Qili Qiangxin Capsule, Qiangxin capsule, Xinlishen compound, Astragalus Granule combined with WM were more effective than WM alone in reducing BNP levels, with statistically significant differences (*P* < .05). Among the comparisons of traditional Chinese medicines, the results showed that Astragalus Granule combined with WM were more effective than Guizhi Yangxin capsule and Xinlishen compound combined with WM, with statistically significant differences (*P* < .05).

**Figure 7.**
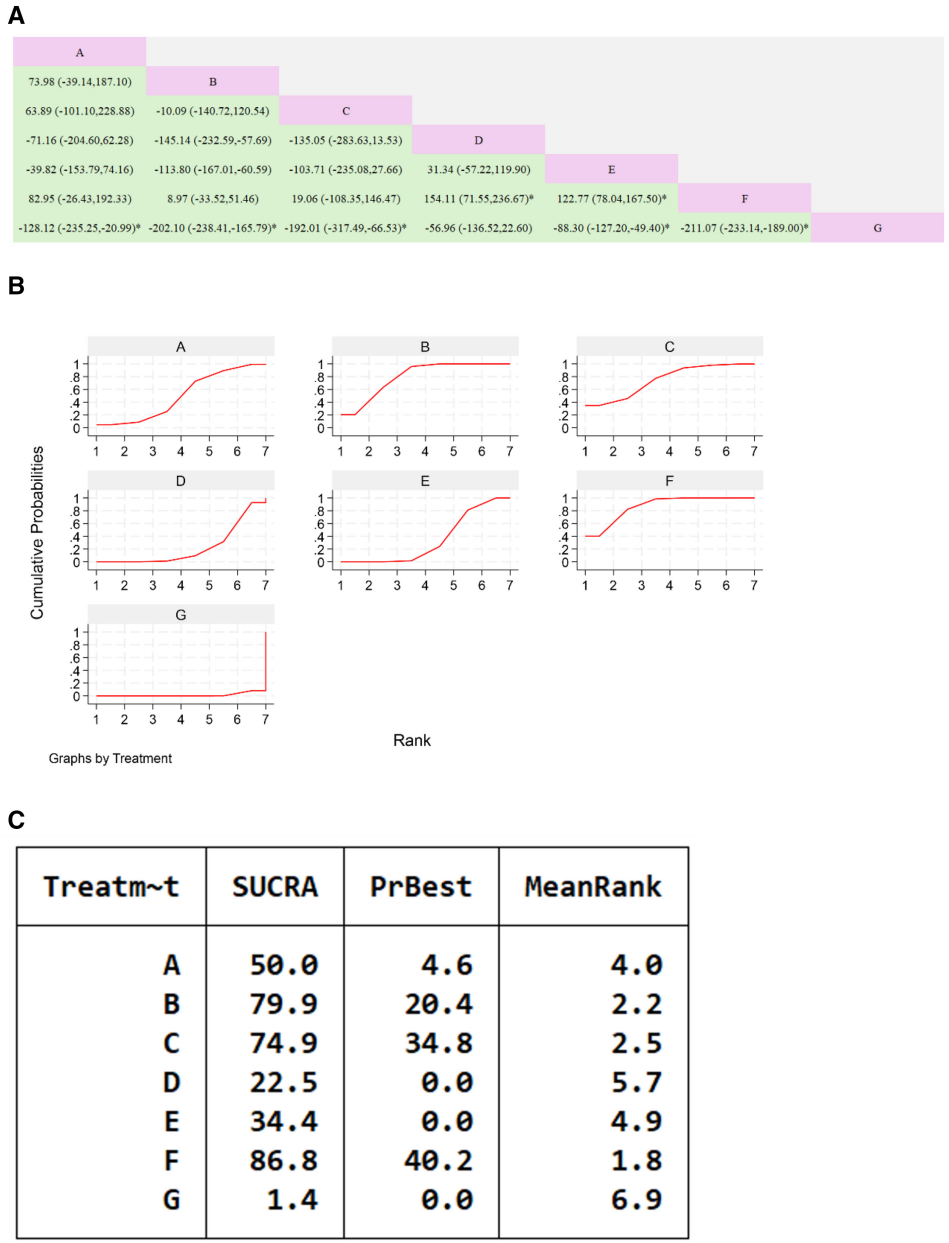
Network meta-analysis of Chinese patent medicines containing Astragalus membranaceus improving BNP levels in patients with heart failure. (A) The league table results of the pairwise network comparisons for improvements in BNP; (B) SUCRA plot of changes in BNP; (C) surface under the cumulative ranking. (A) Yixin Paste; (B) Qili Qiangxin Capsule; (C) Qiangxin Capsule; (D) Guizhi Yangxin Capsule; (E) Xinlishen Compound; (F) Astragalus Granule; (G) WM. BNP = B-type natriuretic peptide, SUCRA = Surface Under the Cumulative Ranking Curve, WM = Western medicine.

The cumulative ranking probability diagram shows that the specific ranking of SUCRA for Chinese patent medicines containing Astragalus membranaceus in improving BNP levels is as follows: Astragalus Granule + WM > Qili Qiangxin Capsule + WM > Qiangxin Capsule + WM > Yixin Paste + WM > Xinlishen Compound + WM > Guizhi Yangxin Capsule + WM > WM, as shown in Figure 7.

#### 3.7.4. N-terminal pro-brain natriuretic peptide

Seven studies reported on NT-proBNP, involving 7 traditional Chinese medicine formulations, with a total of 611 patients. The results of the network meta-analysis showed that the combination of Qili Qiangxin Capsule, Yangxin Tongmai II Prescription, Qiangxinkechuan Decoction, Yiqi Qiangxin Liquid with WM treatment was more effective than using WM alone, with statistically significant differences (*P* < .05); comparisons of traditional Chinese medicines showed that the combination of Qili Qiangxin Capsule with WM was more effective in reducing NT-proBNP levels than the combination of Qiangxinkechuan Decoction, Astragalus Tetrandrae Decoction plus Ephedra, Yiqi Qiangxin Liquid with WM. Yangxin Tongmai II Prescription combined with WM was more effective than Qiangxinkechuan Decoction, Astragalus Tetrandrae Decoction plus Ephedra combined with WM, and Yiqi Qiangxin Liquid combined with WM was more effective than Astragalus Tetrandrae Decoction plus Ephedra combined with WM, with statistically significant differences (*P* < .05). No other statistically significant differences were observed.

**Figure 8.**
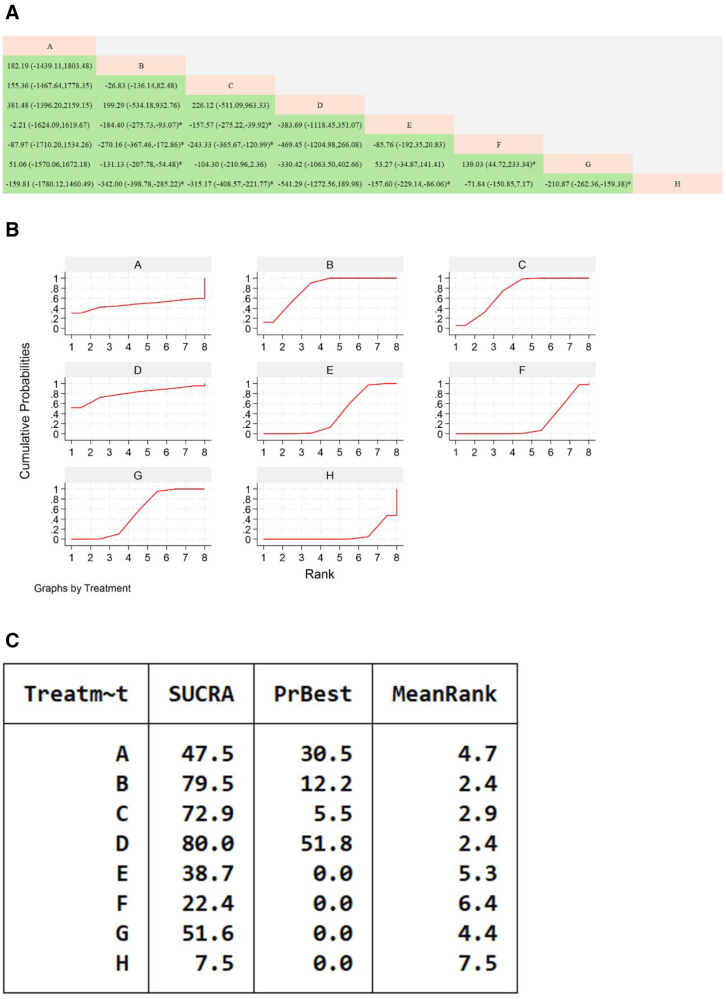
Network meta-analysis of Chinese patent medicines containing Astragalus membranaceus reducing NT-proBNP levels in patients with heart failure. (A) The league table results of the pairwise network comparisons for improvements in NTpro-BNP; (B) SUCRA plot of changes in NTpro-BNP; (C) surface under the cumulative ranking. (A) Xinlishen Compound; (B) Qili Qiangxin Capsule; (C) Yangxin Tongmai II Prescription; (D) Yangxin Decoction; (E) Qiangxinkechuan Decoction; (F) Astragalus Tetrandrae Decoction plus Ephedra; (G) Yiqi Qiangxin Liquid; (H) WM. NT-proBNP = N-terminal pro-brain natriuretic peptide, SUCRA = Surface Under the Cumulative Ranking Curve, WM = Western medicine.

Cumulative ranked probability plot showing the specific ranking of SUCRA for traditional Chinese medicine containing Astragalus membranaceus in improving NT-proBNP levels: Yanxin Decoction + WM > Qili Qiangxin Capsule + WM > Yangxin Tongmai II Prescription + WM > Yiqi Qiangxin Liquid + WM > Xinlishen Compound + WM > Qiangxinkechuan Decoction + WM > Astragalus Tetrandrae Decoction plus Ephedra + WM > WM, as shown in Figure 8.

#### 3.7.5. Left ventricular ejection fraction

Eleven studies reported LVEF, involving 8 types of traditional Chinese medicines and a total of 827 patients. The results of the network meta-analysis showed that the combination of Xinlishen Compound and WM was more effective than WM alone in improving LVEF, with a statistically significant difference (*P* < .05). Other comparisons, including those between traditional Chinese medicines, were not statistically significant.

**Figure 9.**
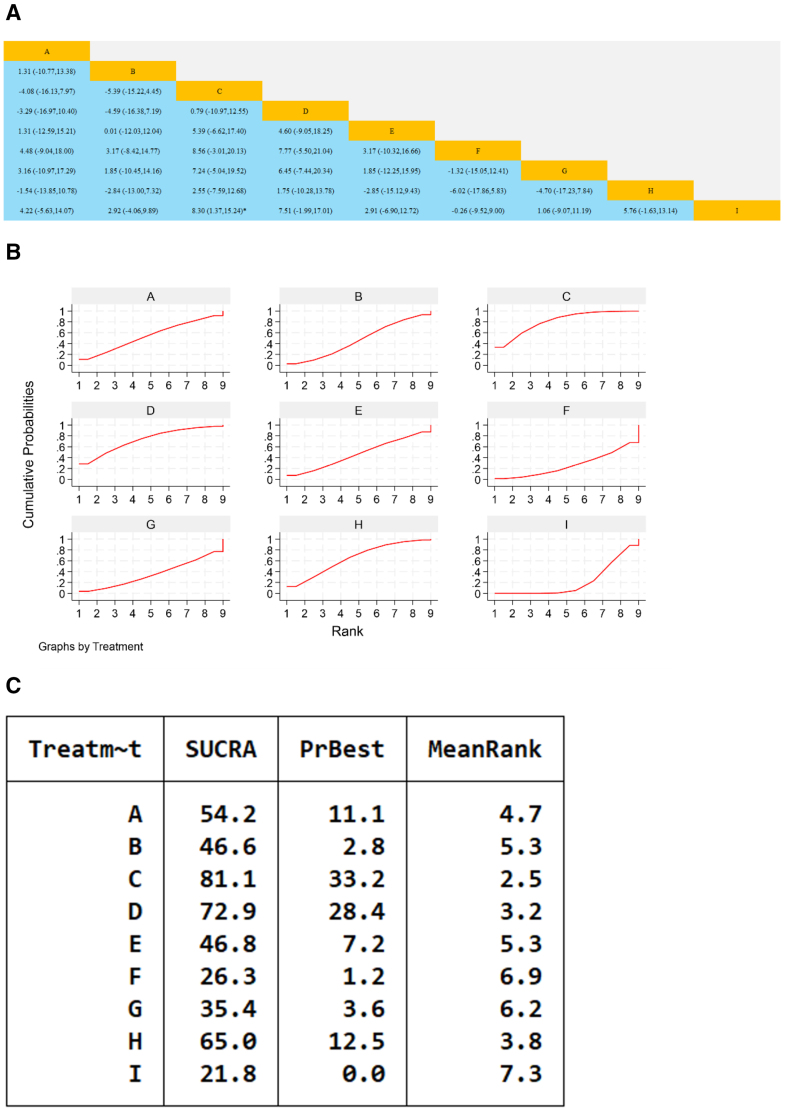
Network meta-analysis of Chinese patent medicines containing Astragalus membranaceus improving LVEF levels in patients with heart failure. (A) The league table results of the pairwise network comparisons for improvements in LVEF; (B) SUCRA plot of changes in LVEF; (C) surface under the cumulative ranking. (A) Yixin Paste; (B) Qili Qiangxin Capsule; (C) Xinlishen Compound; (D) Qiangxin Capsule; (E) Yangxin Decoction; (F) Qiangxinkechuan Decoction; (G) Guizhi Yangxin Capsule; (H) Astragalus Granule; (I) WM, LVEF = left ventricular ejection fraction, SUCRA = Surface Under the Cumulative Ranking Curve, WM = Western medicine.

Cumulative ranked probability plot showing the specific ranking of SUCRA for TCM containing Astragalus in improving LVEF levels: Xinlishen Compound + WM > Qiangxin Capsule + WM > Astragalus Granule + WM > Yixin Paste + WM > Yangxin Decoction + WM > Qili Qiangxin Capsule + WM > Guizhi Yangxin Capsule + WM > Qiangxinkechuan Decoction + WM > WM, as shown in Figure 9.

#### 3.7.6. Minnesota Heart Failure Quality of Life Questionnaire

Three studies reported on MLHFQ, involving 3 types of traditional Chinese medicines, with a total of 316 patients. The results of the network meta-analysis showed that the combination of Shengcao Tongmai granules and Yangxin Tongmai II Prescription with WM was more effective than WM alone in improving MLHFQ, with a statistically significant difference (*P* < .05). Other comparisons between traditional Chinese medicines were not statistically significant.

**Figure 10.**
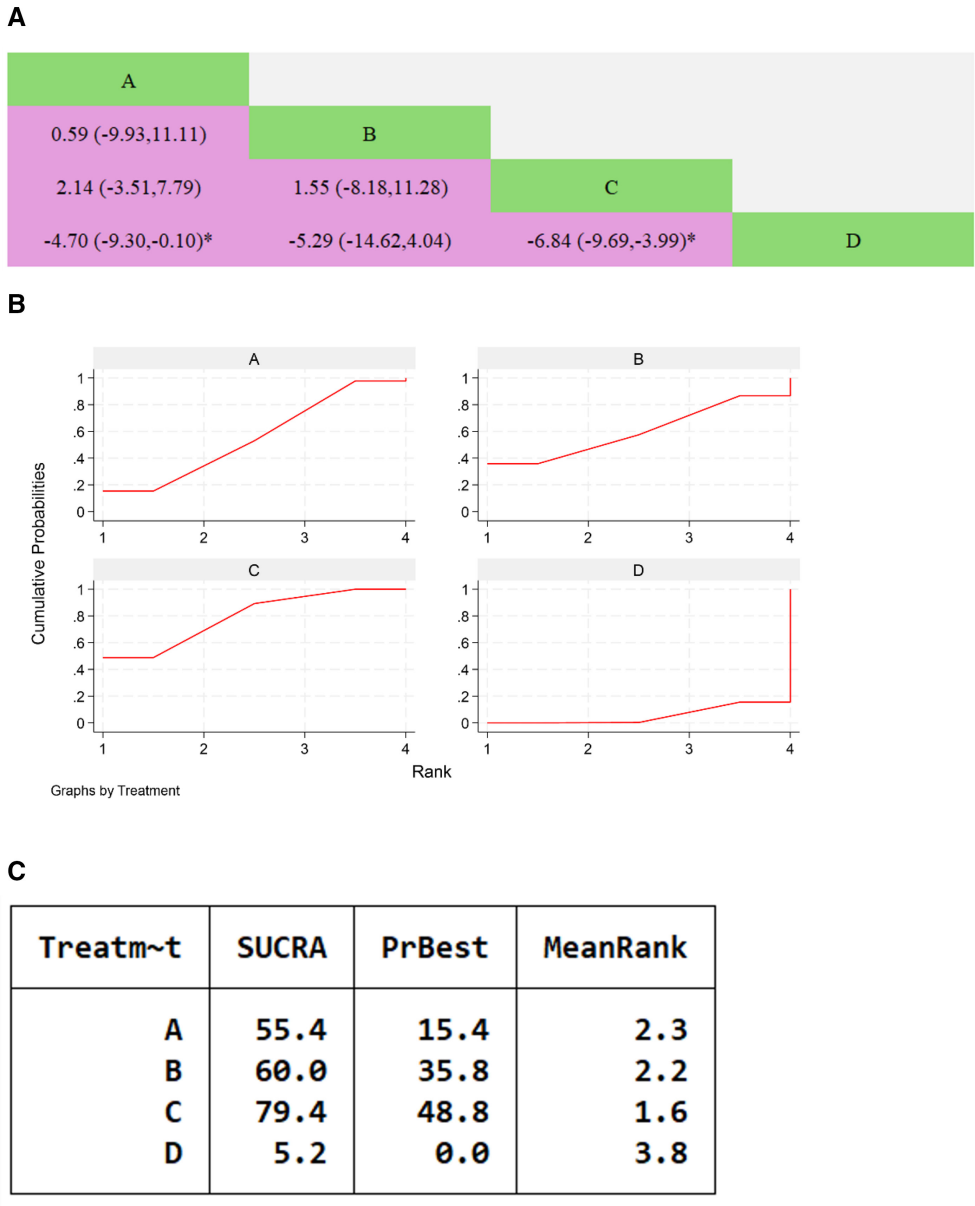
Network meta-analysis of Chinese patent medicines containing Astragalus membranaceus improving MLHFQ in patients with heart failure. (A) The league table results of the pairwise network comparisons for improvements in MLHFQ; (B) SUCRA plot of changes in MLHFQ; (C) surface under the cumulative ranking. (A) Shengcao Tongmai Granules; (B) Danqi baoxinfang; (C) Yangxin Tongmai II Prescription; (D) WM. MLHFQ = Minnesota Heart Failure Quality of Life Questionnaire, SUCRA = Surface Under the Cumulative Ranking Curve, WM = Western medicine.

Cumulative ranking probability diagram: the specific ranking of SUCRA for traditional Chinese medicines containing Astragalus membranaceus in improving MLHFQ is as follows: Yangxin Tongmai II Prescription + WM > Danqi baoxinfang + WM > Shengcao Tongmai Granules + WM > WM, as shown in Figure 10.

#### 3.7.7. Left ventricular end-diastolic diameter

Four studies reported on LVEDD, involving 3 types of traditional Chinese medicines, with a total of 334 patients. The results of the network meta-analysis showed that the combination of Qili Qiangxin Capsule with WM was more effective than WM alone in improving LVEDD, with a statistically significant difference (*P* < .05). Other comparisons, including those between traditional Chinese medicines, were not statistically significant.

**Figure 11.**
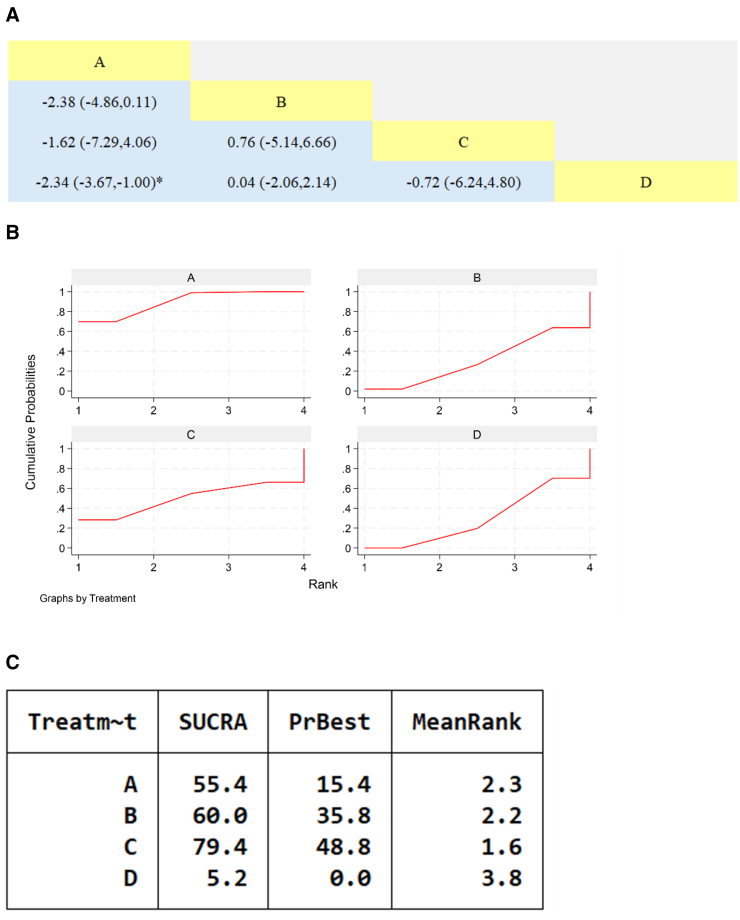
Network meta-analysis of Chinese patent medicines containing Astragalus membranaceus improving LVEDD levels in patients with heart failure. (A) The league table results of the pairwise network comparisons for improvements in LVEDD; (B) SUCRA plot of changes in LVEDD; (C) surface under the cumulative ranking. (A) Qili Qiangxin Capsule; (B) Yanxin Decoction; (C) Guizhi Yangxin Capsule; (D) WM, LVEDD = left ventricular end-diastolic diameter, SUCRA = Surface Under the Cumulative Ranking Curve, WM = Western medicine.

The cumulative ranking probability diagram shows that the specific SUCRA ranking of Chinese patent medicines containing Astragalus membranaceus for improving LVEDD levels is as follows: Qili Qiangxin Capsule + WM > Guizhi Yangxin Capsule + WM > Yanxin Decoction + WM > WM, as shown in Figure 11.

## 4. Discussion

HF in traditional Chinese medicines falls under the categories of “palpitations,” “anxiety,” “dyspnea,” “edema,” and “water retention.” In terms of etiology, it includes external pathogenic factors, emotional trauma, overwork, drug-induced damage, irregular diet, dampness retention, and constitutional deficiency. These are closely related to factors such as infection-induced heart failure, dual heart disease, and increased cardiac volume load in modern medicine. traditional Chinese medicines posits that the fundamental pathogenesis is characterized by “deficiency at the root and excess at the branch,” with deficiency of heart qi and yang as the root, and blood stasis, fluid retention, and phlegm-dampness as the branch. The pathological changes primarily involve the heart, lung, spleen, and kidney organs.^[30]^ Traditional Chinese medicines diagnostic analysis includes: qi and yin deficiency syndrome, heart and kidney yang deficiency syndrome, qi deficiency and blood stasis syndrome, and yang deficiency with water retention syndrome.

Traditional Chinese medicines treatment for HF emphasizes the principle of “treating the symptoms in acute cases and addressing the root cause in chronic cases.” During the acute phase, treatment focuses on diuresis to reduce edema and promoting blood circulation to resolve stasis. During the remission phase, the emphasis shifts to tonifying the heart and kidneys, and nourishing qi and yin.^[31]^ Astragalus, as a key tonic herb for qi, has the effects of tonifying qi, solidifying the exterior, and promoting diuresis to reduce swelling. Modern pharmacological studies have confirmed its significant cardiovascular protective effects,^[32]^ hence it is often used as the primary herb in formulas for treating HF.

Network meta-analysis is an extension of traditional meta-analysis. This study was conducted within a Bayesian framework, first performing a network meta-analysis on oral traditional Chinese medicines containing Astragalus. The results showed that in terms of improving the overall clinical efficacy rate, the combination of Astragalus Granule (an oral medication containing Astragalus) plus WM was the most effective. Astragalus Granule, as a single-ingredient formulation made from Astragalus, not only have immune-modulating effects but also reduce inflammatory factor levels^[33,34]^; in terms of improving the 6MWT, the combination of the traditional Chinese medicines Qiangxin Capsules plus WM yielded the best results, followed by Astragalus Granule plus WM. Qiangxin Capsule primarily consist of Astragalus, Radix Aconiti Lateralis, Radix Sun-dried Ginseng, and Cinnamomi Ramulus, among other herbs, and this medication is widely used in the clinical treatment of HF. Astragalus and Radix Sun-dried Ginseng, act as the primary herbs to tonify qi, while Cinnamomi Ramulus acts as the secondary herb to promote the flow of qi in the heart. Together, these herbs exert a blood-activating and stasis-resolving effect on heart failure^[35]^; in terms of lower BNP levels, Astragalus Granule plus WM showed the best efficacy; in terms of reducing NT-proBNP levels, the Yanxin Decoction plus WM yielded the best results. This formulation consists of Astragalus, Codonopsis pilosula, Angelicae Sinensis Radix, Rhizome of Chuanxiong, Fuling, and hemixia. Astragalus and Codonopsis pilosula serve as the primary herbs, while Rhizome of Chuanxiong, Safflower, Ziziphi Spinosae Semen, and Schisandra chinensis function as secondary herbs to promote blood circulation and qi flow. The combination of these herbs achieves the effects of tonifying qi, promoting blood circulation, nourishing blood, replenishing yin, and calming the mind; in terms of improving LVEF, the Xinlishen Compound plus WM yields the best results. This medication is composed of Astragalus, Codonopsis pilosula, Ophiopogon japonicus, Fuling, Salvia miltiorrhiza, Schisandra chinensis, and Cinnamomi Ramulus eta. In this formula, Astragalus, which is sweet and warm, serves as the principal herb for tonifying the spleen, promoting diuresis, and reducing edema. Panax ginseng, which is sweet and neutral, acts as the auxiliary herb. When used together, these herbs exert the effects of warming yang, tonifying qi, resolving stasis, and promoting diuresis; in terms of reducing MLHFQ, the results of the network meta-analysis showed that the Yangxin Tongmai II Prescription plus WM was the most effective. This formula consists of Codonopsis pilosula, Ophiopogon japonicus, Polygonatum odoratum, Paeonia veitchii Lynch, Salvia miltiorrhiza, roasted licorice, Schisandra chinensis, Aucklandia lappa Decne, and Astragalus. In this formula, Codonopsis pilosula and Astragalus tonify heart qi, Ophiopogon japonicas, Schisandra chinensis, and Polygonatum odoratum nourish qi, generate body fluids, and nourish yin, Aucklandia lappa Decne promotes qi circulation, and Paeonia veitchii Lynch and Salvia miltiorrhiza activate blood circulation, remove blood stasis, and unblock the channels. The entire formula has the effects of tonifying qi, nourishing yin, activating blood circulation, and unblocking the channels.^[36]^ In recent years, pharmacological studies have shown that blood-activating drugs such as Astragalus, Angelicae Sinensis Radix, and Salvia miltiorrhiza can improve vascular endothelial cell dysfunction by regulating nitric oxide utilization. At the same time, Yangxin Tongmai II Prescription can inhibit rat myocardial cell apoptosis and improve the development of HF symptoms. Its active ingredients can improve cardiac function, inhibit cell apoptosis, and delay myocardial remodeling^[37–39]^; in terms of improving LVEDD, the results of the network meta-analysis showed that the combination of Qili Qiangxin Capsule and WM was the most effective. The formula consists of Astragalus, Aconiti Lateralis Radix Praeparata, Panax ginseng, Descurainiae Semen Lepidii Semen, Salvia miltiorrhiza, Safflower and Polygonatum odoratum eta. Among these, Astragalus and Aconiti Lateralis Radix Praeparata serve as the primary herbs to warm yang and tonify qi, while Salvia miltiorrhiza and Descurainiae Semen Lepidii Semen act as auxiliary herbs to promote blood circulation, resolve stasis, and diuretic. The entire formula exerts the effects of warming yang, transforming qi, promoting blood circulation, and resolving stasis. Modern pharmacological studies have shown that Qili Qiangxin Capsule can inhibit myocardial apoptosis, reduce excessive autophagy in myocardial cells by regulating the PI3K/Akt/FoXO3 axis, and effectively improve cardiac function in myocardial ischemia mice through the endoplasmic reticulum stress pathway, while also reducing the infarct area. Astragaloside IV and ginsenoside Rb1 are the main active components in this formula.^[40–43]^

This study has certain limitations: first, none of the 18 studies described the allocation concealment, which may lead to publication bias; the funnel plot results indicate that a small number of samples are at risk of bias, which may lead to lower certainty of evidence for the reported treatment effects; second, the sample sizes of the various oral traditional Chinese medicines were inconsistent, and the evidence map did not form a closed loop, which may affect the stability and reliability of the results; third, all literature was in Chinese, potentially introducing regional and ethnic biases. Therefore, it is recommended that future studies prioritize multicenter randomized controlled trials, emphasize the implementation of blinding and allocation concealment, and incorporate studies with limited evidence to assess efficacy, thereby further enhancing the stability of comparisons between drugs.

## 5. Conclusion

In summary, the combination of oral traditional Chinese medicines containing Astragalus membranaceus with WM significantly improves the overall clinical efficacy rate of HF, enhances LVEF, and reduces levels of BNP, NT-proBNP, LVEDD, and the MLHFQ compared to WM alone. However, some outcome measures showed some bias due to the limited number of included studies. Clinicians should interpret the results of this study with caution and select appropriate oral traditional Chinese medicines formulations based on individual patient conditions during actual treatment. Given the current limitations, future studies will use high-quality, multi-center study outcomes to validate the final results.

## Acknowledgments

We extend our gratitude to the authors of all eligible studies included in this network meta-analysis for their valuable contributions.

## Author contributions

**Conceptualization:** Fangfang Rui, Yan Wu.

**Data curation:** Fangfang Rui.

**Formal analysis:** Fangfang Rui, Yunfeng Di.

**Funding acquisition:** Chun Li.

**Investigation:** Fangfang Rui.

**Methodology:** Fangfang Rui.

**Project administration:** Fangfang Rui, Yan Wu.

**Resources:** Fangfang Rui.

**Software:** Fangfang Rui.

**Supervision:** Fangfang Rui, Yunfeng Di, Chun Li, Tianhua Liu, Yan Wu.

**Validation:** Fangfang Rui, Yan Wu.

**Visualization:** Fangfang Rui, Yunfeng Di, Chun Li, Tianhua Liu.

**Writing – original draft:** Fangfang Rui.

**Writing – review & editing:** Fangfang Rui, Yunfeng Di, Tianhua Liu.

## References

[1] Heidenreich PA Bozkurt B Aguilar D. 2022 AHA/ACC/HFSA Guideline for the Management of Heart Failure: executive summary: a report of the American College of Cardiology/American Heart Association joint committee on clinical practice guidelines. J Am Coll Cardiol. 2022;79:1757–80. 35379504 10.1016/j.jacc.2021.12.011

[2] McDonagh TA Metra M Adamo M; ESC Scientific Document Group. 2023 focused update of the 2021 ESC Guidelines for the diagnosis and treatment of acute and chronic heart failure. Eur Heart J. 2023;44:3627–39. 37622666 10.1093/eurheartj/ehad195

[3] Savarese G Becher PM Lund LH Seferovic P Rosano GMC Coats AJS. Global burden of heart failure: a comprehensive and updated review of epidemiology. Cardiovasc Res. 2023;118:3272–87. 35150240 10.1093/cvr/cvac013

[4] Khan MS Shahid I Bennis A Rakisheva A Metra M Butler J. Global epidemiology of heart failure. Nat Rev Cardiol. 2024;21:717–34. 38926611 10.1038/s41569-024-01046-6

[5] Arnold SV. Assessment of the patient with heart failure symptoms and risk factors: a guide for the non-cardiologist. Diabetes Obes Metab. 2023;25(Suppl 3):15–25. 37337752 10.1111/dom.15166

[6] Chen Z Liu L Gao C. Astragali Radix (Huangqi): a promising edible immunomodulatory herbal medicine. J Ethnopharmacol. 2020;258:112895. 32330511 10.1016/j.jep.2020.112895

[7] Zhang C Zhu Y Wang M Chen R Sun X. Comparison of chemical composition between imitation wild and transplanted Astragali Radix and their therapeutic effects on heart failure. J Ethnopharmacol. 2025;337(Pt 1):118827. 39293703 10.1016/j.jep.2024.118827

[8] Xu X Yang Y Zhou G. Clinical efficacy of Qili Qiangxin Capsule combined with western medicine in the treatment of chronic heart failure: a systematic review and meta-analysis. Evid Based Complement Alternat Med. 2021;2021:9761159. 34408783 10.1155/2021/9761159 PMC8367493

[9] Xia M. Research progress of traditional Chinese medicine in the treatment of chronic heart failure. Mod J Integr Tradit Chin West Med. 2016;25:2278–80.

[10] Wang J Xiong X Feng B. Effect of astragaloside IV on heart failure: a systematic review and meta-analysis of randomized controlled trials. Phytother Res. 2021;35:1304–15.

[11] Brignardello-Petersen R Guyatt GH. Introduction to network meta-analysis: understanding what it is, how it is done, and how it can be used for decision-making. Am J Epidemiol. 2025;194:837–43. 39108176 10.1093/aje/kwae260 PMC11879513

[12] Gao Y Wei FQ Lü XH. Safety evaluation of Yixintong combined with Western medicine in the treatment of chronic congestive heart failure. Da Jia Jian Kang (Xue Shu Ban). 2013;7:17–8.

[13] Cheng WJ Xi JJ Li J. Clinical observation of Qili Qiangxin Capsule in the treatment of elderly patients with chronic heart failure. Hebei J Tradit Chin Med. 2016;38:927–9.

[14] Li QN. Clinical study of Shencao Tongmai Granules in the treatment of chronic heart failure of qi deficiency, blood stasis and water retention type [master’s thesis] [China (Liaoning)]. Liaoning Univ Tradit Chin Med;2012.

[15] Liu GM. The changes of the variability of heart rate in patients with chronic heart failure and the effect of traditional Chinese drug [master’s thesis] [China (Fujian)]. Fujian Coll Tradit Chin Med;2003.

[16] Xiao JH. Analysis of clinical pharmacological effects of Chinese medicine Astragalus membranaceus in the treatment of heart failure. North Pharm. 2024;21:120–2.

[17] Zuo JG. Clinical observation of Yiqiqiangxin fluid on the treatment of 30 patients with chronic heart failure. Proc 4th Acad Conf Emerg Spec Comm World Assoc Tradit Chin Med. 2017:244–52.

[18] Feng QT. Qiliqiangxin capsule in the treatment of chronic congestive heart failure clinical observation and on the B-type natriuretic peptide intervention of clinical research. Hebei Med Univ. 2013.

[19] Li YY. Clinical study of Qiangxin Capsule in the treatment of elderly CHF (heart-kidney yang deficiency type) and its effect on BNP [master’s thesis] [China (Yunnan)]. Yunnan Coll Tradit Chin Med. 2016.

[20] Pan QJ. Clinical study of modified Yangxin Decoction in the treatment of CHF of qi-yin deficiency and blood stasis type with Yanxin Decoction [master’s thesis] [China (Jiangxi)]. Jiangxi Univ Tradit Chin Med. 2021.

[21] Yang QY Lu S. Sun HR effect of Astragalus membranaceus on cardiac function and serum tumor necrosis factor level in patients with chronic heart failure. Chin J Integr Tradit West Med. 2010;30:699–701. 20929124

[22] Zhao ZX. Clinical observation on Astragalus Tetrandrae Decoction plus Ephedra in treatment of chronic cardiac failure with water overflowing caused by insufficiency of yang [master’s thesis] [China(Guangxi)]. Guangxi Univ Tradit Chin Med. 2018.

[23] Chen SH Liu Z Wu HR. Yixin paste in the treatment of 30 cases of chronic heart failure. Shanxi J Chin Tradit Med. 2009;30:647–8.

[24] Song FL. Clinical study on the therapeutic effect of Guizhi YangXin Capsule in the treatment of chronic congestive heart failure [master’s thesis] [China (Gansu)]: Gansu Coll Tradit Chin Med. 2014.

[25] Wei HY Yang QY Wang YF Gong SY. Effect of Xinlishen on plasma BNP, AngII and cardiac function in patients with congestive heart failure. Shanxi J Chin Tradit Med. 2008:653–5.

[26] Huang Y. Effect of Xinlishen on heart failure echocardiography index and Tei index in patients with heart qi(yang) deficiency type of chronic heart failure. Nanjing Univ Tradit Chin Med. 2018.

[27] Lu J. Clinical study of Yangxin Tongmai II Prescription in the treatment of chronic heart failure of Chronic Heart Failure with Deficiency of Qi and Yin [master’s thesis] [China (Fujian)]. Guangxi Univ Tradit Chin Med. 2019.

[28] Qu Y. A clinical study on the effect of qiangxinkechuan decoction in treating patients with type heart and kidney Yang deficiency chronic heart failure on decompensation. [master’s thesis] [China (Shandong)]. Shandong Univ Tradit Chin Med. 2013.

[29] Li XP. A experiment and clinical research on the ventricular remodeling of chronic heart failure treating by Danqi Baoxin Formula [master’s thesis] [China (Chengdu)]. Chengdu Univ Tradit Chin Med. 2014.

[30] Lan Y Luo FK Yu Y Wang XY Wang PQ Xiong XJ. Coronary heart disease: innovative understanding from traditional Chinese medicine and treatment by classic formulas. Zhongguo Zhong Yao Za Zhi. 2024;49:3684–92. 39041141 10.19540/j.cnki.cjcmm.20240326.501

[31] Hao P Jiang F Cheng J Ma L Zhang Y Zhao Y. Traditional Chinese medicine for cardiovascular disease: evidence and potential mechanisms. J Am Coll Cardiol. 2017;69:2952–66. 28619197 10.1016/j.jacc.2017.04.041

[32] Yao J Peng T Shao C Liu Y Lin H Liu Y. The antioxidant action of astragali radix: its active components and molecular basis. Molecules. 2024;29:1691. 38675511 10.3390/molecules29081691 PMC11052376

[33] Yang XG Hao S Yuan J. Huangqi particle with western medicine on diabetic retinopathy complicated with diabetic nephropathy. Shanxi J Chin Tradit Med. 2015;36:1477–9.

[34] Liu CH Abrams ND Carrick DM. Imaging inflammation and its resolution in health and disease: current status, clinical needs, challenges, and opportunities. FASEB J. 2019;33:13085–97. 31577913 10.1096/fj.201902024 PMC6894066

[35] Luo SY. Clinical observation of Qiangxin Capsule combined with sacubitril/valsartan in patients with hypertension complicated with HFpEF [master’s thesis] [China (Yunnan)]. Yunnan Univ Tradit Chin Med;2023.

[36] Luo WK Lu JQ Lu J Tang ML Peng ZL Dong L. Effect of Yangxin Tongmai Formula Ⅱ on cardiac function and endothelial function in patients with chronic heart failure of qi-yin deficiency and blood stasis type. Glob Tradit Chin Med. 2024;17:1650–4.

[37] Cao PX Ge LD Wang DC Wu H. An overview of the mechanism of TCM of Yiqi Huoxue for the treatment of ischemic heart disease by improving endothelial cell damage. Glob Tradit Chin Med. 2023;16:2365–70.

[38] He XB Wen ZH Liu XL Pan CX Wang QG Huang MJ. Effects of Yangxin Tongmai Mixture on cardiac function and neuroendocrine factor of patients with chronic heart failure. China Contemp Med. 2017;24:140–2.

[39] Xu L He D Wu Y Shen L Wang Y Xu Y. Tanshinone IIA inhibits cardiomyocyte apoptosis and rescues cardiac function during doxorubicin-induced cardiotoxicity by activating the DAXX/MEK/ERK1/2 pathway. Phytomedicine. 2022;107:154471. 36182795 10.1016/j.phymed.2022.154471

[40] Wu N Chi J Cai H. Traditional Chinese medication qili qiangxin capsule protects against myocardial ischemia-reperfusion injury through suppressing autophagy via the phosphoinositide 3-kinase/protein kinase B/forkhead box O3 axis. J Ethnopharmacol. 2025;337(Pt 1):118821. 39265794 10.1016/j.jep.2024.118821

[41] Ji XD Yang D Cui XY. Mechanism of Qili Qiangxin capsule for heart failure based on miR133a-endoplasmic reticulum stress. Chin J Integr Med. 2024;30:398–407. 38386253 10.1007/s11655-024-3654-3

[42] He L Liu Y Yang K. The discovery of Q-markers of Qiliqiangxin Capsule, a traditional Chinese medicine prescription in the treatment of chronic heart failure, based on a novel strategy of multi-dimensional "radar chart" mode evaluation. Phytomedicine. 2021;82:153443. 33429210 10.1016/j.phymed.2020.153443

[43] Zhang F Zhang Y Li X. Research on Q-markers of Qiliqiangxin capsule for chronic heart failure treatment based on pharmacokinetics and pharmacodynamics association. Phytomedicine. 2018;44:220–30. 29699844 10.1016/j.phymed.2018.03.003

